# Yellow Lamb Disease (*Clostridium perfringens* Type A Enterotoxemia of Sheep): A Review

**DOI:** 10.3390/ani12121590

**Published:** 2022-06-20

**Authors:** Francisco A. Uzal, Federico Giannitti, Javier Asin

**Affiliations:** 1 California Animal Health and Food Safety, University of California-Davis, San Bernardino, CA 92408, USA; jasinros@ucdavis.edu; 2 Plataforma de Investigación en Salud Animal, Instituto de Investigación Agropecuaria, Estación Experimental La Estanzuela, Colonia 70000, Uruguay; fgiannitti@yahoo.com

**Keywords:** alpha toxin, *Clostridium perfringens* type A, icterus, yellow lamb disease

## Abstract

**Simple Summary:**

Yellow lamb disease is a poorly researched and understood condition that seems to affect young sheep. The disease is characterized by hemolysis and is thought to be caused by alpha toxin-producing *Clostridium perfringens* type A, although a definitive association with this microorganism has not been confirmed. This is due, in part, to the ubiquitous nature of *C. perfringens* type A, which is naturally present in the intestine of healthy sheep, a fact that complicates the diagnosis. In this review, we summarize the available information on the etiology, clinical signs, lesions, diagnosis, prevention and prophylaxis of yellow lamb disease.

**Abstract:**

Yellow lamb disease is an infrequent disease in sheep for which there is scant literature, and that has been reported in the US, Australia, New Zealand, South Africa and Europe, although anecdotal evidence indicates that it may have also been diagnosed in South America. The disease is produced by some strains of *Clostridium perfringens* type A that produce unusually high levels of alpha- toxin. Because *C. perfringens* type A is ubiquitous and is found in the intestine of most clinically healthy sheep, diagnosis of yellow lamb disease is challenging and requires quantitating the amount of this microorganism present in feces and/or intestinal content. Clinically, yellow lamb disease is characterized by depression, anemia, icterus and hemoglobinuria. Occasionally, sudden death may occur. Gross findings include generalized icterus, red urine in the bladder, enlarged, pale, and friable spleen, enlarged liver with an acinar pattern, and dark, swollen kidneys. Microscopically, yellow lamb disease is characterized by centrilobular necrosis of the liver, hemoglobinuria-associated acute tubular injury, splenic congestion, pulmonary congestion and edema. Although there are no vaccines specifically designed to prevent yellow lamb disease, several vaccines against the different types of *C. perfringens* may afford at least some level of protection against yellow lamb disease.

## 1. Introduction

Yellow lamb disease, also known as type A enterotoxemia or enterotoxemic jaundice, is a rare disease in sheep, thought to be caused by *Clostridium perfringens* type A, which is poorly characterized. The condition has been described in Australia, New Zealand, South Africa, the USA and Europe [1,2,3], with anecdotal evidence suggesting that cases might have occurred in South America (authors’ unpublished observations). Because *C. perfringens* type A is found in the intestine of many clinically healthy animal species, including sheep [3], the diagnosis of the disease is challenging as isolation of this microorganism has little, if any, diagnostic significance. It is therefore possible that the occurrence of yellow lamb disease may be underestimated in several parts of the world. Here, we review the literature on yellow lamb disease. Electronic databases including PubMed, CAB Abstracts and Google Scholar were screened for literature without time restrictions using the following search terms: “*Clostridium perfringens* type A”, “*Clostridium perfringens* alpha toxin”, “lamb”, “sheep”, “yellow lamb disease”, “enterotoxemic jaundice”, “type A enterotoxemia” and combinations thereof. Additionally, the reference lists of relevant publications obtained from this initial search were carefully screened for additional relevant publications. The database from the California Animal health and Food Safety laboratory (1990–2022) was also searched for cases of yellow lamb disease.

## 2. Etiology and Pathogenesis

**Figure 1:**
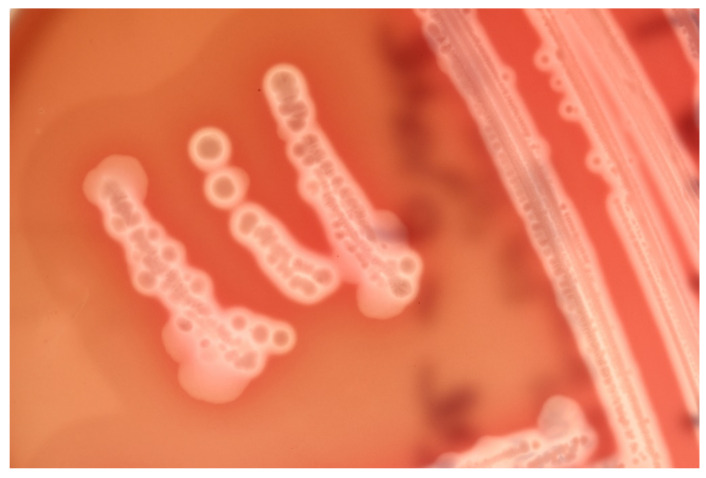
Colonies of *Clostridium perfringens* on blood agar, surrounded by two halos of hemolysis including an inner, complete zone of hemolysis caused by perfringolysin and an outer zone caused by alpha toxin.

*C. perfringens* is classified into seven toxinotypes (A, B, C, D, E, F and G) based on the production of six so-called major toxins, namely alpha (CPA), beta (CPB), epsilon (ETX), iota (ITX), enterotoxin (CPE) and necrotic enteritis toxin B-like (Table 1) [4]. In addition, different strains of *C. perfringens* can produce close to 20 so-called minor toxins in various combinations. Morphologically, the colonies and cells of all the toxinotypes are identical. After 24 h of incubation under anaerobic conditions in blood agar, the colonies are usually 3–5 mm in diameter, circular and grayish. In most cases, two halos of hemolysis surround the colonies, i.e., an inner, complete zone of hemolysis that is caused by perfringolysin, one of the so-called minor toxins produced by this microorganism, and an outer incomplete halo that is caused by CPA (Figure 1) [5]. There is a small percentage of *Clostridium perfringens* strains that does not carry the perfringolysin gene, so these strains may not show the characteristic double hemolytic zone that is normally seen.

Most enteric *C. perfringens* infections are called enterotoxemias. These are conditions characterized by production of toxins in the intestine. The toxins are then absorbed into the systemic circulation and exert their effects in distant organs. *C. perfringens* type A has been suggested to be implicated in enterotoxemia of several animal species [5,6], but evidence-based information to support this claim is scant or missing. The possible exception for the previous statement is yellow lamb disease [1,2,3], a rare form of enterotoxemia observed mostly, but not exclusively, in lambs, and allegedly caused by certain strains of *C. perfringens* type A that may produce unusually large amounts of CPA [3]. Most of the information available in the scientific literature on the cause and pathogenesis of yellow lamb disease has been generated through observational studies of spontaneous field outbreaks. To the best of our knowledge, the disease has not been experimentally reproduced in ruminants, and Koch postulates have not been fulfilled, which represents a major knowledge gap. Information about the pathogenesis of this disease is minimal and often contradictory, but it is generally assumed that most clinical signs and lesions are due to the effects of CPA. An outbreak of what was thought to be yellow lamb disease has been reported in lambs infected by a strain of *C. perfringens* type D that produced an unusually high amount of CPA [7]. CPA is a highly hemolytic lecithinase (phospholipase), which is thought to be responsible for the severe hemolysis and jaundice observed in lambs with yellow lamb disease. The hemolytic activity of CPA on various animal species has considerable variation, with sheep erythrocytes being the most sensitive to the action of this toxin [8].

All strains of *C. perfringens* carry the gene encoding CPA, located in a stable region of the chromosome [9,10,11]. CPA is a zinc metalloenzyme composed of 370 amino acids that binds to host cell membranes in the presence of calcium ions [9,12]. The toxin is composed of two main domains: the catalytic N-domain, and the membrane binding C-domain. CPA has also a central loop domain containing a ganglioside binding site or GM1a [9,12,13].

**Figure 2:**
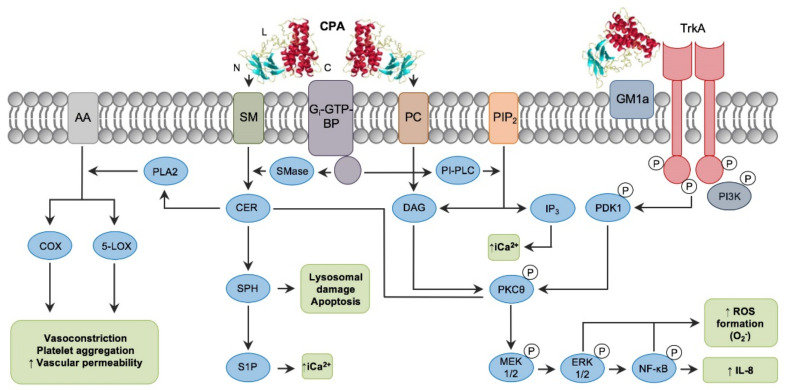
Intracellular pathways involved in *C. perfringens* alpha toxin (CPA) intracellular action. Note the binding C-domain (C), catalytic N-domain (N) and ganglioside-binding loop domain (L) of CPA (PDB ID: 1CA1). CPA directly hydrolyzes phosphatidylcholine (PC) and sphingomyelin (SM) present in the plasma membrane of target cells. CPA can also activate G_i_-type GTP-binding protein (G_i_-GTP-BP) present in the plasma membrane, which in turn will activate endogenous phospholipases (PI-PLC) and sphingomyelinases (SMase). Phospholipase activity results in the formation of diacylglycerol (DAG), and inositol trisphosphate (IP3); the latter mobilizes and increases intracytoplasmic calcium ions (_i_Ca^2+^). Sphingomyelinase action results in ceramide (CER), sphingosine (SPH) and sphingosine-1-phosphate (S1P) formation. In addition, interaction of CPA with the TrkA receptor leads to PDK1 and PKCθ phosphorylation, resulting in activation of the MEK/ERK signaling cascade and NF-κB, which is involved in ROS and IL-8 formation (Reprinted with permission from Navarro et al., 2018 [9]).

Several different pathways are affected during CPA action on cell membranes (Figure 2), where this toxin hydrolyzes phosphatidylcholine (PC) to sphingomyelin (SM), producing diacylglycerol and ceramide, respectively [9]. CPA also indirectly activates endogenous host enzymes, which have similar phospholipase and sphingomyelinase properties. CPA binding to cell membranes also leads to the activation of the MEK/ERK pathway. Synergism between CPA and sialidases has been suggested, as sialic acids are important in the structure of gangliosides. The removal of sialic acid by *C. perfringens* sialidases increased cell sensitivity to CPA in vitro and in vivo [14] Synergism between CPA and perfringolysin also occurs in cases of human gas gangrene [3].

CPA binds to and acts on membrane PC and SM, in addition to activating G_i_ type GTP-binding proteins (G_i_-GTP-BPs), triggering different pathways depending on the cell type involved (Figure 2). For instance, sheep erythrocytes are affected by CPA mainly by activation of the SM metabolism via GTP-BPs [8,15]. The mechanism of cell death promoted by lytic amounts of CPA involves extensive damage of plasma membrane and lactate dehydrogenase release, which is characteristic of necrosis [16]. However, sub-lytic concentrations of CPA are associated with the activation of the MEK/ERK pathway and the generation of reactive oxygen species, which can lead to oxidative stress and activate intrinsic mechanisms of apoptosis [17].

## 3. Epidemiology

Reports of yellow lamb disease that include clinical and epidemiological data are very scarce in the scientific literature. It is generally accepted that the disease occurs most commonly in young animals, although it does not seem to be exclusively restricted to this age group. McGowan et al. [1] described the disease in suckling lambs in California, and reports from other geographical areas seem to restrict the age range to young individuals as well, but also include fattening lambs and yearlings [18]. Conversely, some of the first reported cases of yellow lamb disease in Australia occurred in mature ewes, wethers and rams, but not in lambs or yearlings. Based on this observation, the authors suggested that there was an apparent increment of susceptibility with age [2]. Because of this, the colloquial name yellow lamb disease can be misleading given that the condition may also affect older sheep. The only outbreak of yellow lamb disease associated with a type D strain reported occurred in 3- to 4-week-old lambs [7].

Risk factors associated with yellow lamb disease are unknown, although factors that predispose to other types of enterotoxemia, such as a sudden dietary change to a high carbohydrate ration could play a role [3]. The early outbreaks described in California in the fifties occurred over a 6-week period in the spring; the affected lambs and their mothers were on native pasture in 4/6 outbreaks and on improved pasture in 2/6 outbreaks. Rainfall was above normal and green watery feed was abundant [1]. The outbreak associated with the type D strain in California occurred in early spring, although information on the type of diet the animals were receiving was unavailable (authors unpublished observation). Outbreaks in Australia have occurred in semi-drought conditions as well as on flush pastures in every month of the year, with no seasonal incidence [2].

Rose et al. [2] in Australia observed that the disease was mostly restricted to British breeds of sheep, with Merino rarely becoming affected even when exposed to similar risk factors. The outbreak associated with *C. perfringens* type D referred to above occurred in Katahdin lambs [7], but the breed was not reported in the earlier publication describing six outbreaks in California [1]. To our knowledge there are no solid epidemiological studies assessing whether breed is a significant risk factor for yellow lamb disease.

The reported mortality of type A enterotoxemia varies from 4% (of 2400 lambs over a 6-week period on six ranches in California) [1], to 55% (550/1000 ewes during 9 months in Australia) [2]. In the outbreak associated with a type D strain of *C. perfringens*, mortality was 3.5%, affecting 7 of 200 lambs in one flock in California [7]. Lethality is thought to be very high.

## 4. Clinical Signs

A rapid clinical course is usually described, with lambs found dead or surviving no more than 12 h after the onset of clinical signs [1]. Icteric conjunctiva and sclera, increased heart and respiratory rates, weakness, anemia with blood packed cell volume as low as 4%, hemoglobinuria, red-tinged feces and abdominal pain are clinical signs described in affected lambs [1,5,7].

## 5. Gross Changes

**Figure 3:**
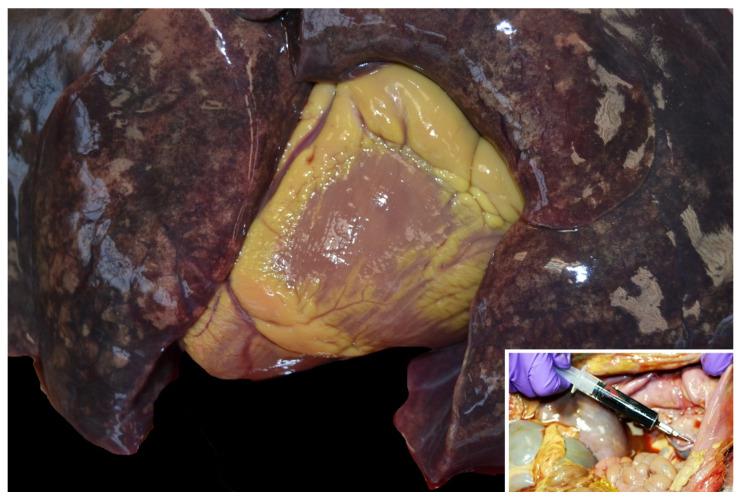
Marked yellow discoloration of the epicardial fat of a lamb with icterus. Inset: Dark red urine in a lamb with hemoglobinuria. Note diffuse icterus in abdominal and pelvic cavity tissues.

**Figure 4:**
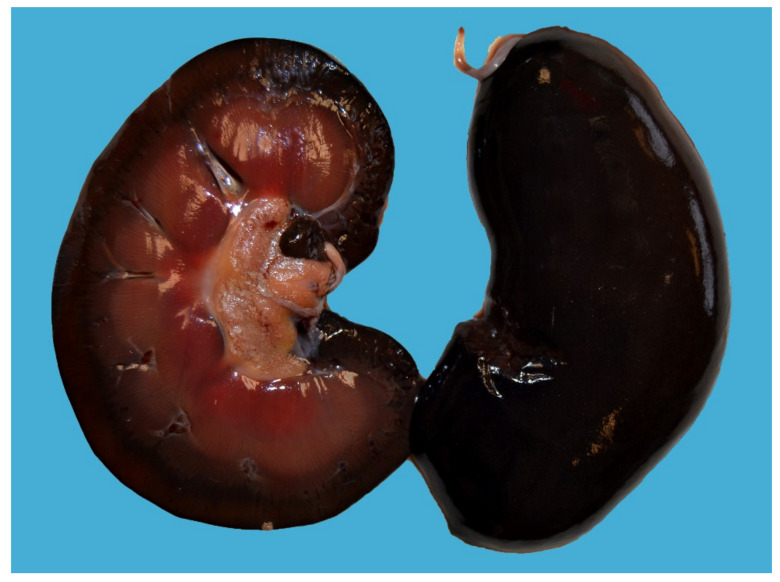
Diffuse dark red to brown discoloration of the renal cortex and medulla in a lamb with hemoglobinuria.

**Figure 5:**
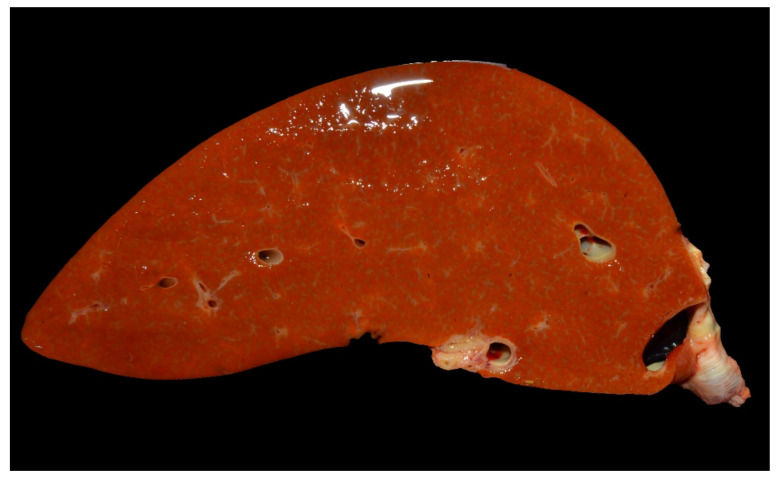
Yellowish, enlarged liver in a lamb with marked icterus.

Lesions of type A enterotoxemia have seldomly been described in the scientific literature [1,2,19]. Gross lesions are those typically present in cases of acute intravascular hemolysis of any cause. Icterus is a constant finding (Figure 3), and gives rise to the colloquial name, yellow lamb disease, although the mucous membranes can be pale due to anemia. Occasionally, lamb carcasses have been condemned at the slaughterhouse due to icterus and this has been suggested to be associated with yellow lamb disease, although such diagnoses were not confirmed (19. Hemoglobinemia and hemoglobinuria most notably result in diffuse, reddish-brown discoloration of the kidneys and urine (Figure 3 and Figure 4), but a pink discoloration of the tissues and body fluids can also occur. Enlarged, pale and/or slightly yellowish and friable liver has also been described in a large proportion of cases (Figure 5) [1]. Less constant findings include pulmonary edema and reddening with pink stable froth in the lumen of the bronchi/trachea, and petechiae in the endocardium, epicardium, or peritoneum [1]. In the only report of yellow lamb disease attributed to *C. perfringens* type D [7], which produces CPA and ETX (Table 1), lesions were similar to those described for type A. However, in that case, pulmonary edema was striking and there was also red-tinged subcutaneous edema and accumulation of pink serous fluid in the thoracic cavity [7]. It was speculated that such lesions could be mediated by the action of ETX on the vascular endothelium resulting in increased vascular permeability [20]. That case also had moderate enlargement of the spleen [7]. No gross lesions were observed in the spleen of earlier cases described in California [1] or Australia [2].

## 6. Microscopic Changes

**Figure 6:**
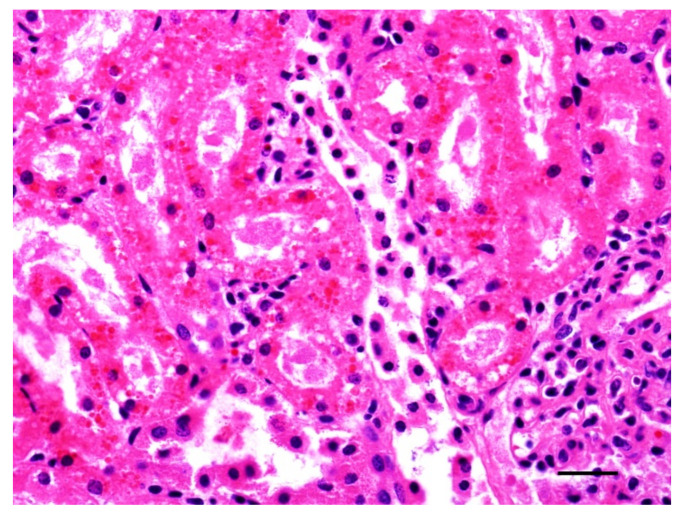
Kidney of a lamb with yellow lamb disease showing hemoglobinuria-associated acute tubular injury. The renal cortical tubular epithelial cells are laden with variably-size, round, eosinophilic intracytoplasmic hyaline protein droplets. Hematoxylin and eosin. Bar = 60 µm.

**Figure 7:**
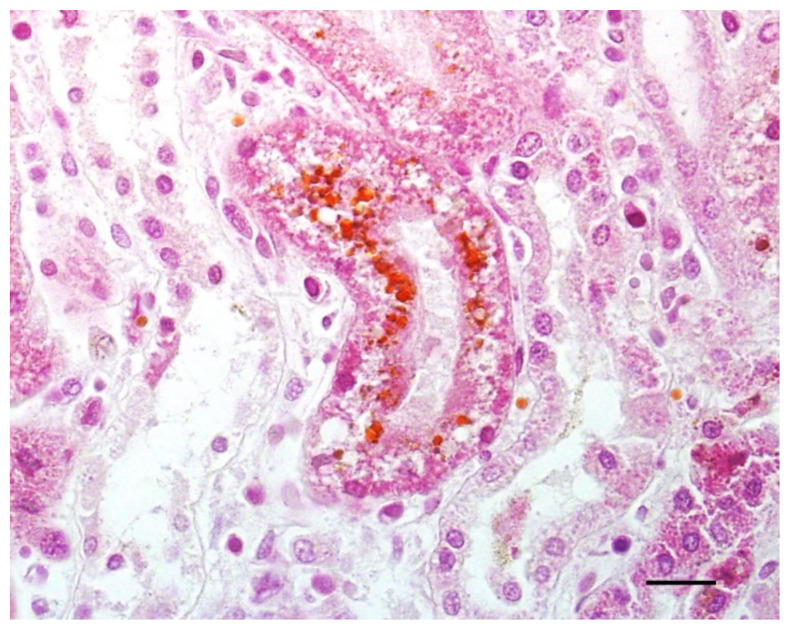
Kidney of a lamb with yellow lamb disease showing hemoglobinuria-associated acute tubular injury. The intracytoplasmic droplets are stained orange/brown with an Okajima stain for hemoglobin. Bar = 40 µm.

**Figure 8:**
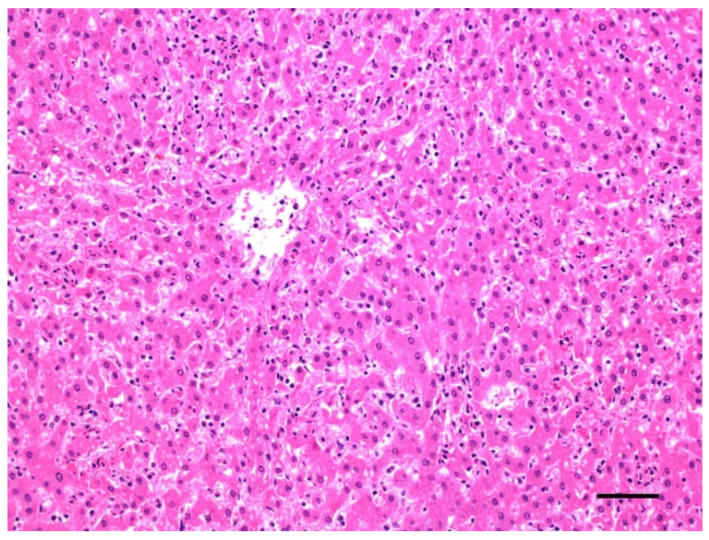
Liver of a lamb with yellow lamb disease, showing marked centrilobular degeneration. Hematoxylin and eosin. Bar = 100 µm.

Histologically, hemoglobinuria-associated acute tubular injury (formerly known as pigmentary or hemoglobinuric nephrosis) is the hallmark of renal lesions (Figure 6). The cortical tubular epithelial cells show varying degrees of swelling, intracytoplasmic hyaline droplets, necrosis, and sloughing. These changes affect most notably the proximal and, to a lesser extent, the distal convoluted tubules [1]. Protein casts are usually seen in the lumen of affected tubules. The intracytoplasmic hyaline droplets and intratubular casts can be highlighted with Okajima stain for hemoglobin (Figure 7) [7]. Centrilobular hepatocellular swelling, vacuolation, and necrosis is a common finding [1], and it is likely a result of hypoxia secondary to hemolysis (Figure 8). A direct effect of CPA on hepatocytes is also possible, although this has not been proved. Although McGowan et al. [1] indicated that there was no excess bile pigment in the canaliculi or bile ducts, bile stasis in the liver has been described in early reports of the disease [2], as well as in the case associated with *C. perfringens* type D [7]. The histologic correlate of pulmonary reddening is hyperemia [1]. Pulmonary edema usually involves the alveolar spaces and the interstitium [7]. A slight increase in red blood cells in the red pulp is the only documented histologic change in the spleen [1].

## 7. Diagnosis

No definitive diagnostic criteria have been established for yellow lamb disease. A presumptive diagnosis is usually made based on clinical, gross and microscopic findings, coupled with ruling out other causes of intravascular hemolysis. *C. perfringens* CPA is neither the only nor the most common cause of acute intravascular hemolysis in sheep. Thus, while icterus, hemoglobinuria-associated acute tubular injury, and hemoglobinuria are consistent with yellow lamb disease, they are by no means confirmatory. Differential diagnoses include other causes of hemolysis, such as copper toxicosis, leptospirosis, hemotropic mycoplasmosis (formerly known as eperithrozoonosis) and hemoparasites such as *Anaplasma ovis*, amongst others [5,21,22,23,24].

There are no laboratory tests available to confirm a diagnosis of yellow lamb disease. Although detection of *C. perfringens* type A and CPA in intestinal content or feces is compatible with a diagnosis of this disease, the mere detection of either one or both of them cannot be considered diagnostic. The reason is that both *C. perfringens* type A and CPA can be found with high prevalence in the intestine of healthy sheep. It has been suggested that the presence of 10^4^ to 10^7^ colony forming units of *C. perfringens* type A per gram of intestinal content is diagnostic for yellow lamb disease [5,21]. However, this has not been confirmed and it remains speculative. In addition, colony counts are usually not performed routinely in veterinary diagnostic laboratories. This is compounded by the fact that there is little information available about the number of *C. perfringens* type A cells in the intestine of healthy and sick animals. Establishing a threshold for this count in sick animals would be helpful to refine the diagnostic criteria for yellow lamb disease in the future. The use of real-time PCR to quantify the load of *C. perfringens* (and/or the CPA encoding gene) could shed some light on this issue in years to come. Similarly, although CPA is frequently found in the intestine of normal sheep, no information about the amount of this toxin present in healthy or diseased animals is available. The fact that most enzyme-linked immunosorbent assays used routinely to detect *C. perfringens* toxins are highly sensitive and can detect minimal concentrations of CPA, adds another layer of complication to the diagnostic process. Developing and validating toxicologic assays for the routine quantification of CPA and other *C. perfringens* toxins in intestinal contents in diagnostic settings could be of value in this regard. Over the past few years, several diagnostic laboratories have started offering PCR to detect the genes of some of the typing toxins of *C. perfringens*, including CPA. This method has the same limitations explained above for the isolation of *C. perfringens* type A, as the gene encoding CPA can be found in the intestine of most normal animals, associated with this or other toxinotypes of *C. perfringens* [5,21].

While detection of the microorganism and/or CPA is not diagnostic for yellow lamb disease, failure to detect them in the intestine of affected sheep helps in ruling out a diagnosis of this disease.

## 8. Prophylaxis, Control and Treatment

Data about prophylaxis and control of type A enterotoxemia are very limited, as expected for a disease with largely unknown predisposing factors. Assuming that similar risk factors as for other types of enterotoxemia such as type D may play a role, the same preventive measures should be applied [3]. This is, avoiding animals being suddenly exposed to starch-rich diets, combined with vaccination. Many vaccines developed for different types of *C. perfringens* contain also inactivated CPA [25], and therefore should offer a degree of protection for type A enterotoxemia. All this, however, remains speculative. Anecdotally, one affected lamb that was given blood transfusions survived type A enterotoxemia [1], but in general no treatment methods for yellow lamb disease have been described to the best of our knowledge. Considering the very acute nature of the disease, treatment options are limited.

## 9. Conclusions

Yellow lamb disease seems to be a rare condition on which there are scant descriptions in the scientific literature. This lack of information might be associated with the low prevalence of the disease, lack of accurate diagnostic tests and criteria, or a combination of both. One of the main problems for the diagnosis of this disease is that it is believed to be produced by *C. perfringens* type A, which is the most common toxinotype found in the intestine of normal sheep. Something similar happens with CPA, which is also present in the intestine of normal animals. Distinguishing normal microbiota from pathogenic strains is always challenging. Determining the threshold at which either *C. perfringens* type A and/or CPA result in clinical disease would be a useful step towards establishing diagnostic criteria for yellow lamb disease. Finally, it is also possible that certain type A strains produce still unknown toxins that may have a primary or secondary role in the pathogenesis of yellow lamb disease. Experimental reproduction of the disease in ruminants to fulfill Koch’s postulates would help to better understand the pathogenesis of this largely under-researched condition.

## Figures and Tables

**Table 1:** Classification of *Clostridium perfringens* (Rood et al., 2018 [4]).

*C. perfrigens* Type	Typing Toxins					
	CPA	CPB	ETX	ITX	CPE	NetB
A	+	−	−	−	−/+	−
B	+	+	+	−	−/+	−
C	+	+	−	−	−/+	−
D	+	−	+	−	−/+	−
E	+	−	−	+	−/+	−
F	+	−	−	−	+	−
G	+	−	−	−	−	+

CPA: Alpha toxin; CPB: Beta toxin; ETX: Epsilon toxin; ITX: Iota toxin; CPE: Enterotoxin; NetB: Necrotic enteritis B-like toxin.

## Data Availability

Not applicable.
